# Practical Approach to Eliminate Solidification Cracks by Supplementing AlMg4.5Mn0.7 with AlSi10Mg Powder in Laser Powder Bed Fusion

**DOI:** 10.3390/ma15020572

**Published:** 2022-01-13

**Authors:** Constantin Böhm, Martin Werz, Stefan Weihe

**Affiliations:** Materials Testing Institute (MPA), University of Stuttgart, Pfaffenwaldring 32, D-70569 Stuttgart, Germany; stefan.weihe@mpa.uni-stuttgart.de

**Keywords:** laser powder bed fusion, solidification crack, hot crack, aluminum alloy powder, EN AW-5083, AA5083, AlSi10Mg, selective laser melting

## Abstract

The range of available aluminum alloy powders for laser powder bed fusion (LPBF) is restricted to mainly Al–Si based alloys. Currently aluminum alloy powders, designed for lightweight application, based on Al–Mg (5000 series), Al–Si–Mg (6000 series), or Al–Zn–Mg (7000 series), cannot be processed by LPBF without solidification cracks. This has an impact on the potential of LPBF for lightweight applications. In fusion welding, solidification cracks are eliminated by using filler materials. This study aims to transfer the known procedure to LPBF, by supplementing EN AW-5083 (AlMg4.5Mn0.7) with AlSi10Mg. EN AW-5083 and two modifications (+7 wt.% and +15 wt.% AlSi10Mg) were produced by LPBF and analyzed. It was found that, in EN AW-5083, the solidification cracks have a length ≥200 µm parallel to the building direction. Furthermore, the solidification cracks can already be eliminated by supplementing 7 wt.% AlSi10Mg. The microstructure analysis revealed that, by supplementing AlSi10Mg, the melt pool boundaries become visible, and the grain refines by 40% relative to the base alloy. Therefore, adding a low melting point phase and grain refinement are the mechanisms that eliminate solidification cracking. This study illustrates a practical approach to eliminate solidification cracks in LPBF.

## 1. Introduction

Solidification cracks are severe defects that limit the processability of the commercially-available aluminum alloy powders for laser powder bed fusion (LPBF), also known as selective laser melting (SLM) [1]. This hinders the potential of lightweight design with LPBF.

The LPBF process is physically similar to fusion welding. In LPBF, a part is created by iteratively melting metal powder along multiple tracks, with a focused laser beam in a single plane, and adding new powder layers to build up the three-dimensional part. The part is built up on a substrate plate, where the powder is added layer-by-layer by a powder recoater. In contrast to fusion welding, single or multiple seams are welded to join parts.

The LPBF process has numerous influence factors that all impact the quality of the part [2]. Overall, as reviewed in [3], the research in LPBF focusses on residual stresses and distortion, the influence of the process on the surface quality, and their influence on the mechanical properties of the LPBF-produced parts, as well as the interaction of the laser beam with the metal powder [4,5,6]. Another important research subject is the density of the part. Research groups focus on understanding the mechanisms of pore formation [7], in order to find optimal process parameters for multiple materials [8,9]. Furthermore, processing defects, such as splattering [10], balling [11], and disproportionate evaporation of volatile elements [2,12,13], which can degrade the quality of the LPBF-produced part, are addressed in current research. Another research area deals with the influence of the properties of the metal powder on the finished part, as well as the process stability [14,15,16]. In contrast to the process-related research, the LPBF machines are equally subject of a research and development effort [17].

Additional effort is invested into developing high-strength aluminum alloy powders for LPBF, as summarized by Leirmo [1], where solidification cracks must be overcome to ensure their processability with LPBF. Various strategies are used to eliminate or reduce solidification cracks in fusion welding [18]. Solidification cracks can be reduced in fusion welding (i) by controlling the solidification structure by supplementing grain refiner [19,20] or adjusting the process parameters to increase the grain refinement [21] or using specially-designed alloys [22] and (ii) by using favorable welding conditions by adjusting the process parameters to reduce the stress on the weld seam [23] or adapting the joint configuration [18].

These strategies were already applied to eliminate or reduce solidification cracks during the LPBF process. For example, the powder alloy composition of AA7075 (AlZnMg5.5Cu) was controlled by supplementing elemental silicon [24] or AlSi10Mg powder [25] to create a powder–powder mixture. Mechanically mixing metal powders is a novel technique used in LPBF research that enables flexible alloy design. This technique was performed for various metal systems in LPBF [8,24,25,26,27,28,29,30]. In all these studies [8,24,25,26,27,28,29,30], the metal powders were mixed dry for several minutes to hours. There is yet no uniform procedure for mixing the powders. Furthermore, the effects of the mixing parameters on the homogeneity of the resulting powder is still ongoing research [30]. Moreover, the LPBF process parameters were adjusted in [13,31,32,33], and the base plate heating was adjusted in [12] to eliminate or reduce solidification cracks. In addition, the solidification structure was controlled to reduce solidification cracks by adding grain refiner [13,32,34,35,36].

Pre-alloying powder alloys [13,32] and/or coating with nanoparticles [34,36] requires expensive or sophisticated technology and trained staff. In contrast, mixing the base alloy with a supplement powder to modify and control the alloy composition appears to be a practical technique. For example, Aversa et al. [25] supplemented 50 wt.% of AlSi10Mg to AA7075 to eliminate solidification cracks, and Montero-Sistiaga et al. [24] supplemented 4 wt.% elemental silicon to AA7075. However, supplementing too much silicon results in brittle phases that can reduce the tensile ductility. For the 50 wt.% AA7075 and 50 wt.% AlSi10Mg alloy, Aversa et al. [25] found an elongation of break of 2.7%, whereas for AA7075-T6, a typical value is 10% [37]. Therefore, an objective for this research field is to minimize the amount of supplemented AlSi10Mg.

In order to calculate the minimal required AlSi10Mg addition to a base powder alloy with a solidification crack criterion [38,39], the mechanisms that lead to the elimination of solidification cracks need to be understood first. Furthermore, experimental data for low additions of AlSi10Mg is required to validate solidification crack criteria. Up to now, there is still a knowledge gap in current literature.

The 5000 series aluminum alloys show good weldability, good formability, and high-strength, as well as good corrosion resistance [37,40]. One example of the 5000 series is EN AW-5083. The alloy composition is listed in Table 1. Its high magnesium content reduces the density of the alloy, as well as increases its weldability and solid solution strength. Therefore, it is a widely-used alloy for lightweight applications [37,40]. However, solidification cracks form when processing this alloy with LPBF [13]. How solidification cracks in EN AW-5083 can be avoided by supplementing AlSi10Mg has not yet been investigated in literature. There is a lack of experimental data.

The objective of this study is to understand to what extent the selected process parameters can reduce the solidification cracking susceptibility in EN AW-5083 and how even small supplements of AlSi10Mg (+7 wt.% and +15 wt.% AlSi10Mg) can reduce the solidification cracking susceptibility of EN AW-5083. This study provides essential experimental data and insights for computer-aided alloy design.

## 2. Materials and Methods

**Figure 1:**
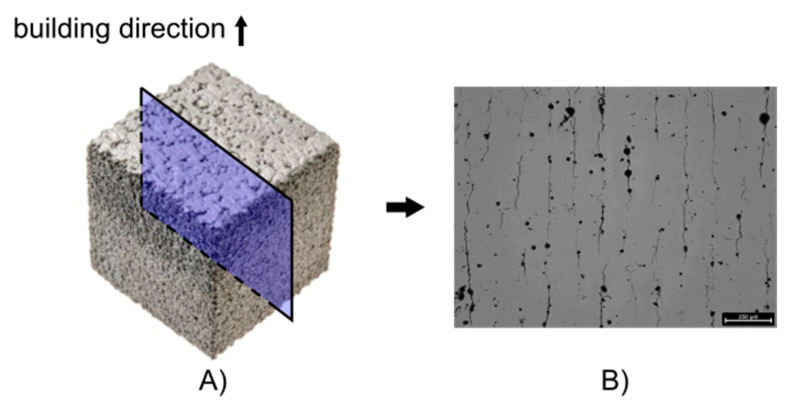
(**A**) Cubical specimens, 10 × 10 × 10 mm^3^, were produced with LPBF and cut perpendicular to the building direction. (**B**) The cross sections were cut, polished, and captured with a light microscope, in order to reveal the solidification cracks parallel to the building direction.

In order to investigate the influence of the amount of supplemented powder and process parameters, cubical specimens (10 × 10 × 10 mm^3^) were manufactured with LPBF and analyzed metallographically, as seen in Figure 1. All specimens were produced with a LPBF system by AconityMINI (Aconity3D GmbH, Herzogenrath, Germany).

The used scan strategy was unidirectional alternating by 90° in every layer. For all conducted experiments, the powder layer thickness was *t* = 30 µm, and no substrate heating was used. The scan velocity (*v* = 500–1500 mm/s) was varied because it is a main influence on the solidification cracking sensitivity, as shown in [38]. Furthermore the focal diameter was adjusted, in order to increase the size of the weld pool, thereby decreasing the expected solidification crack sensitivity [18]. The hatch distance was adjusted according to the focal diameter to get the required overlap of the melt tracks. Table 2 summarizes the process parameters used. Table 2 also highlights the volumetric energy density (*VED*) = *P*/( *v* ∙ *h* ∙ *d_f_*). Overall, the laser power was adjusted to reach a value of *VED*, which led to dense parts made of AlSi10Mg in prior LPBF experiments conducted at the institute.

For each combination of process parameters, one specimen was produced and investigated further.

Two alloy modifications were prepared, in order to investigate the effect of supplementing AlSi10Mg on the solidification cracks. The alloy composition of the base alloys, supplement AlSi10Mg powder, and two modifications are listed in Table 3. The amount of AlSi10Mg was chosen, in order to get double (+7 wt.% AlSi10Mg) and fourfold (+15 wt.% AlSi10Mg) the maximum allowed silicon content in EN AW-5083, according to DIN EN 573-3 [41] (Table 1). The goal was to keep the amount of supplemented AlSi10Mg as low as possible because there is no available data in this region.

**Figure 2:**
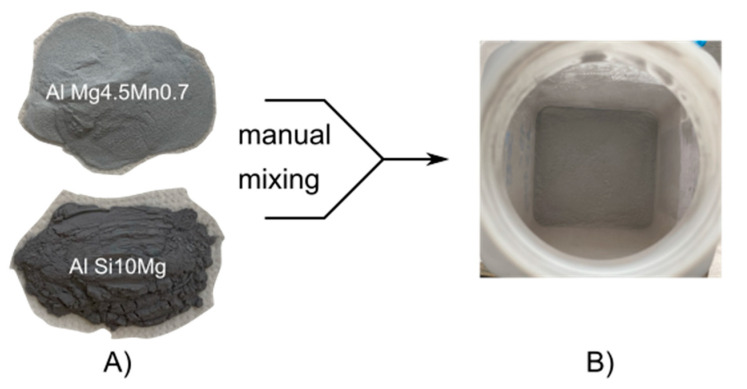
(**A**) Base alloy EN AW-5083 (AlMg4.5Mn0.7) was mixed with the supplement AlSi10Mg powder. (**B**) Homogeneous mixture by manually shaking the two powders in a sealed container.

The modifications were prepared by weighing and manually mixing them, as shown in Figure 2. The base alloy EN AW-5083 (supplied by Rosswag GmbH, Pfinztal, Germany) and supplement AlSi10Mg (supplied by TLS Technik GmbH & Co. Spezialpulver KG, Bitterfeld-Wolfen, Germany) were mixed in a sealed container by manually shaking and rotating it along its longitudinal axis for 30 s. The mixing procedure was repeated five times for each powder mixture. After mixing, the resulting powder blend was inspected by naked eye, in order to qualitatively evaluate the homogeneity of the blend. This evaluation was possible due to the optical difference of AlSi10Mg, which appears darker due to the high silicon content, compared to the base alloy EN AW-5083 (AlMg4.5Mn0.7), which appears brighter under ambient lighting, as shown in Figure 2.

The powder size distribution for EN AW-5083 was D10 = 26.36 µm, D50 = 41.37 µm, and D90 = 62.16 µm for the respective percentile quantile. For AlSi10Mg it was D10 = 14.53 µm, D50 = 30.13 µm, and D90 = 44.14 µm. The powder supplier measured and provided this data with a dynamic image analysis.

The resulting silicon content (Table 3) in the modifications exceeded the allowed silicon content of the EN AW-5083 standard, where a maximum of 0.4 wt.% is specified. The composition of the modifications is comparable to the EN AW-5026 alloy, which has an allowed silicon content of 0.55–1.4 wt.% and magnesium content of 3.9–4.9 wt.% [41]. Due to the increased silicon content, the modifications are considered to be hardenable by Mg_2_Si precipitations.

The LPBF-produced specimens of the base alloy and two modifications were cut, polished, and analyzed with light and electron microscopes. The specimens were polished wet with SiC abrasive paper, with grit sizes in the following sequence: P180, P320, P400, P800, P1200 (European *P*-grade). Afterwards, the specimens were further polished in a sequence with diamond suspension with the following sizes: 15 µm, 6 µm, and 1 µm. Lastly, the specimens were vibration polished with SiO_2_ of size 0.05 µm for approximately two hours for the inspection with the light microscope and approximately 24 h for the inspection with the electron microscope.

The analysis with the light microscope (Leica DM8000M, Leica, Wetzlar, Germany) was conducted for all prepared specimens. The light microscope images were used to approximate the density/porosity of the part. For the calculation of the density, the image processing software ImageJ v1.52a [42] was used. First, the greyscale light microscope image was adjusted to a black-and-white image, with the function *Threshold* of ImageJ. Next, the function *Analyze Particles* was used to calculate the area fraction of the pores. The automatic settings of ImageJ were used for all image processing steps. One image per specimen was analyzed in this way.

The analysis of the microstructure was done with a scanning electron microscope (SEM) (Auriga SEM System, Carl Zeiss AG, Oberkochen, Germany). The analysis was conducted for the base alloy and two modifications prepared with the process parameters B (Table 2). For the microstructure analysis, an electron back scattering diffraction (EBSD), as well as an energy-dispersive X-ray spectroscopy (EDX), was performed. The EDX signal was measured during the EBSD measurement. Therefore, it is to be interpreted qualitatively. The microstructure analysis with SEM was performed with an acceleration voltage of 20 kV at a magnification of ×100 of an 800 × 800 µm region, with a step size of 2 µm. A DigiView detector (EDAX, Mahwah, NJ, USA) was used for EBSD, and an Octane Pro detector (EDAX, Mahwah, NJ, USA) was used for EDX.

## 3. Results

### 3.1. Metallographic Analysis of EN AW-5083 (AlMg4.5Mn0.7)

**Figure 3:**
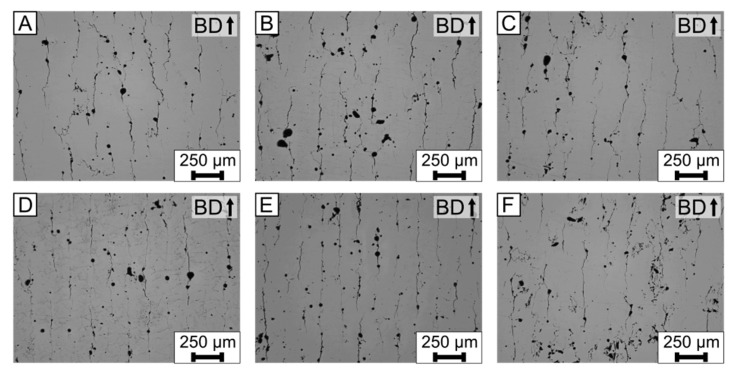
Light microscope images of the polished cross sections for the base alloy EN AW-5083 (AlMg4.5Mn0.7). (**A**–**F**) Process parameters are listed in Table 2 and correspond to the letter of each cross section. Furthermore, the results of the density measurements, which were done with image processing, are listed in Table 4.

The process parameters were varied, in order to evaluate their influence on the solidification cracks in EN AW-5083 (AlMg4.5Mn0.7). Of particular interest was the variation of the scan velocity *v* because it influences the solidification and cooling rates [18]. Both are influence factors on the solidification crack susceptibility [38]. Figure 3 depicts the polished cross sections of the cubic specimens, viewed with the light microscope. The used process parameters are listed in Table 2. The density was calculated by image processing and varied between 95.4–97.5%, as summarized in Table 4.

In all images of Figure 3, cracks parallel to the building direction are present. The crack length is between 100 µm to 300 µm. The horizontal spacing between the cracks is between 50–150 µm. In the chosen process window, the cracks could not be eliminated. However, in Figure 3A,D, the cracks occur qualitatively lesser in frequency and length, compared to the Figure 3C,F.

This seconds the initial assumption that the cracks are solidification cracks. With an increase in the *VED*, the temperature gradient and cooling rate is reduced, which subsequently leads to a reduction of the solidification crack susceptibility [38]. If liquation cracks were present, an increase in the *VED* would lead to a higher crack frequency, due to an increased heat-affected zone [18].

**Figure 4:**
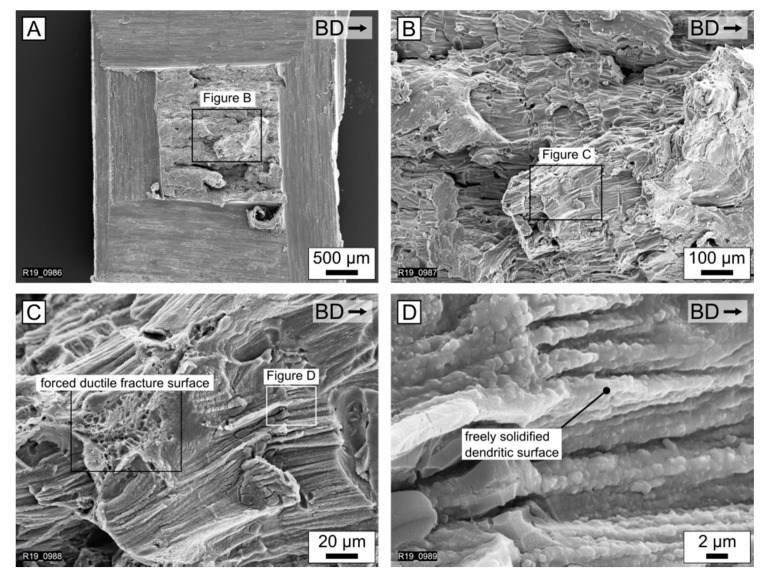
SEM images of the fracture surface of the base alloy EN AW-5083 (AlMg4.5Mn0.7). (**A**) Indicating the procedure for the forced fracture in the laboratory, (**B**) magnification highlighting the melt pools, (**C**) magnification highlighting the forced ductile fracture surface and dendritic structure, and (**D**) magnification of freely-solidified dendritic surface, typically for solidification cracks.

Figure 4 depicts images, captured with SEM, of the fracture surface of an EN AW-5083 specimen. The sample was cut and then forcefully torn apart, in order to reveal the surface of the solidification cracks. Figure 4C shows the residual fracture surface and the freely-solidified dendritic structure. The ductile forced fracture surface is a result of the laboratory procedure to beak the specimen. However, the freely-solidified dendritic structure in Figure 4D is a result of the solidification during the LPBF process.

As a conclusion, the freely-solidified dendritic structure and increase in the *VED*, leading to a reduction in the crack frequency, are evidence for solidification cracks. Furthermore, the variation of the process parameters in the given process window (*VED* = 52 J/mm^3^–25 J/mm^3^) could not eliminate the solidification cracks in the LPBF-produced EN AW-5083 (AlMg4.5Mn0.7) specimens. Kouraytem et al. [43] showed that pores, such as those present in Figure 3, can lead to solidification cracks. Therefore, there could be a combination of process parameters, where no pores or solidification cracks are present. However, this combination of process parameters is currently unknown for EN AW-5083.

### 3.2. Modifying EN AW-5083 (AlMg4.5Mn0.7) with AlSi10Mg

#### 3.2.1. Modification I: AlMg4.5Mn0.7 +7 wt.% AlSi10Mg

**Figure 5:**
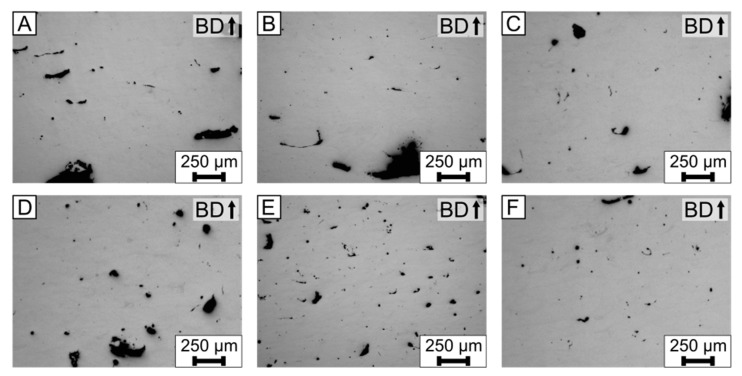
Light microscope images of the polished cross sections for the MOD1 +7 wt.% AlSi10Mg (Table 3). (**A**–**F**) Process parameters are listed in Table 2 and correspond to the letter of each cross section. The results of the density measurements, which were calculated with image processing, are listed in Table 5.

The process parameters were varied, in order to evaluate their influence on the solidification cracks in the modified alloy MOD1 (AlMg4.5Mn0.7 +7 wt.% AlSi10Mg). Figure 5 depicts the polished cross sections of the cubic specimens, viewed with the light microscope. The process parameters used are listed in Table 2. The resulting density (Table 5) of the specimens was measured with image processing and range between 95.3–99.1%.

In all images of Figure 5, no solidification cracks are visible for all combinations of the process parameters. However, there are various processing defects, such as lack of fusion or porosities. Porosities and lack of fusion can be reduced by adjusting the process parameters, for example by increasing the *VED*, as proposed by Weingarten et al. [7] and Ghasemi-Tabasi et al. [9].

Even though pores can initiate solidification cracks [43], no solidification cracks were observed for the modification I with a residual porosity. In conclusion, supplementing AlSi10Mg can eliminate the solidification cracks.

#### 3.2.2. Modification II: AlMg4.5Mn0.7 +15 wt.% AlSi10Mg

**Figure 6:**
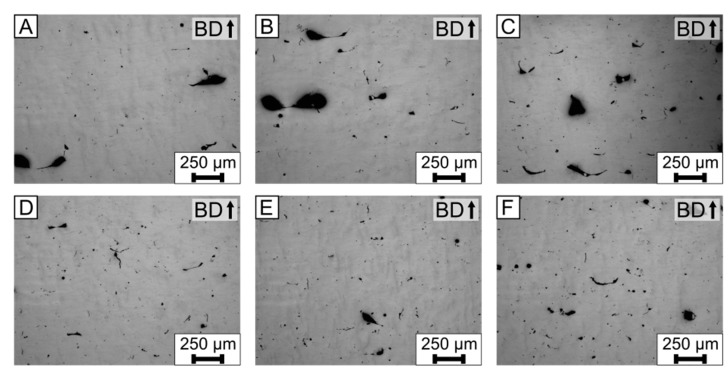
Light microscope images of the polished cross sections for the MOD2 +15 wt.% AlSi10Mg (Table 3). (**A**–**F**) Process parameters are listed in Table 2 and correspond to the letter of each cross section. Table 6 lists the results of the density measurements, which were calculated with image processing.

The process parameters were varied, in order to evaluate their influence on the solidification cracks in the modified alloy MOD2 (AlMg4.5Mn0.7 +15 wt.% AlSi10Mg). Figure 6 depicts the polished cross sections of the cubic specimens, viewed with the light microscope. The process parameters used are listed in Table 2. Table 6 lists the results of the density measurements, which were done with image processing. The density of the MOD2 specimens range between 96.6–99.1% for the used process parameters.

In all images of Figure 6, no solidification cracks are visible for any combination of the process parameters, similar to the results of Modification I (Figure 5). However, single microcracks, with different orientations, are visible in Figure 6B,D,E. Furthermore, there are various processing defects, such as lack of fusion or porosities. Lack of fusion and porosities can be reduced by adjusting the process parameters further.

For both modifications, solidification cracks were eliminated, even though there were pores. As shown by Kouraytem et al. [43], pores can lead to solidification cracks.

### 3.3. Microstructure Analysis

In this section, the microstructure is studied in more detail, in order to highlight the mechanisms that eliminate the solidification cracks. The effect of supplementing AlSi10Mg on the grain structure is demonstrated. Furthermore, the distribution of the alloying elements, compared to the base powder alloy, is analyzed qualitatively.

#### 3.3.1. EBSD Measurement

**Figure 7:**
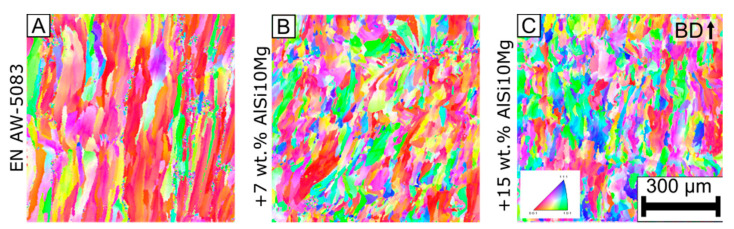
Electron back scattering diffraction (EBSD) for the (**A**) base alloy EN AW-5083, (**B**) MOD1 +7 wt.% AlSi10Mg, and (**C**) MOD2 +15 wt.% AlSi10Mg (Table 3). The process parameters for all specimens were *p* = 250 W, *v* = 1000 mm/s, and *d*_f_ = 100 µm (Table 2).

Figure 7 depicts the EBSD analysis of the base alloy and its two modifications. The analyzed specimens were processed with the process parameters B in Table 2. The colors indicate the different grain orientations. All images are aligned according to their building direction (BD).

In the base alloy EN AW-5083, the grains grow parallel to the building direction. The grains in the base alloy reach lengths between 300–500 µm. There are no melt pool boundaries visible. The grains outgrow multiple layers.

In the modification +7 wt.% AlSi10Mg, the grains grow parallel to the building direction. However, they are smaller, compared to the ones of the base alloy. The melt pool boundaries are visible for the modification +7 wt.% AlSi10Mg. Similar observations can be drawn from the modification +15 wt.% AlSi10Mg.

The EBSD analysis revealed that the base alloy has elongated grains that could lead to the increased solidification crack susceptibility. Supplementing the base alloy with AlSi10Mg leads to a qualitative grain refinement.

**Figure 8:**
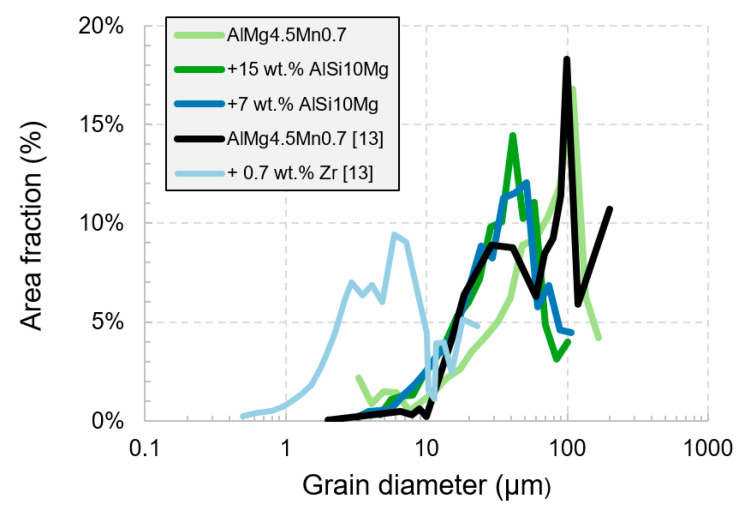
Histogram of the area fraction over the grain diameter. The data was extracted from the EBSD measurement Figure 7.

The grain diameter and area fraction of these grains can be calculated from the EBSD analysis. Figure 8 compares the area fraction of a specific grain diameter for the different alloys in this study with results from literature [13]. The x-axis in Figure 8 represents the grain diameter (µm) in a logarithmic scale. The y-axis represents the area fraction (%) of a specific grain diameter.

The grain diameter with the maximum area fraction for both base alloys is approximately 100 µm. Modifying the base alloy reduces the grain diameter with the maximum area fraction to 40 µm (+15 wt.% AlSi10Mg), 50 µm (+7 wt.% AlSi10Mg), and 6 µm (+0.7 wt.% Zr). Furthermore, by modifying the alloy, the mean grain diameter is reduced, as listed in Table 7. Relative to the base alloy, the mean grain diameter is reduced by 40% by modifying it with +7 wt.% or +15 wt.% AlSi10Mg. Modifying the base alloy with zirconium reduces the mean grain diameter, relative to the base alloy by 91% [13]. Zirconium has a higher potential for grain refinement than AlSi10Mg because it adds more nucleation particles to the alloy.

Supplementing the alloy with AlSi10Mg increases the fraction of dissolved alloying elements in the melt, which subsequently restricts the grain growth and promotes a grain refinement. The grain refinement decreases the solidification cracks significantly. In other studies, grain refiners, such as titanium or zirconium, were supplemented to the base alloy to reduce the solidification cracks [34,36].

#### 3.3.2. Qualitative EDX Measurement

**Figure 9:**
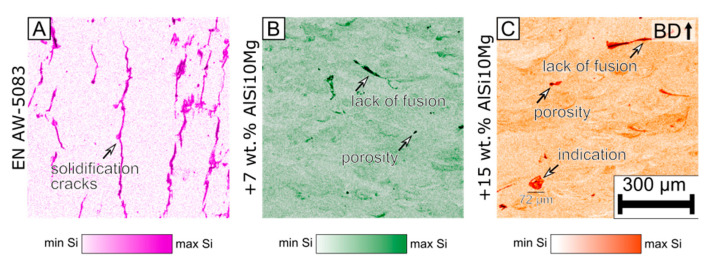
Qualitative energy-dispersive X-ray spectroscopy (EDX) was done during EBSD measurement for the (**A**) base alloy EN AW-5083, (**B**) MOD1 +7 wt.% AlSi10Mg, and (**C**) MOD2 +15 wt.% AlSi10Mg (Table 3). The process parameters for all specimens were *p* = 250 W, *v* = 1000 mm/s, and *d_f_* = 100 µm. (Table 2).

Figure 9 depicts the results of the qualitative EDX measurement. The EDX signal was recorded during the EBSD measurement. In Figure 9, the color bars qualitatively indicate the amount of silicon present in the cross section. The color bars are not comparable with each other. A different color scheme was chosen to avoid misinterpretation. The goal of the EDX measurement was to get an indication of how homogeneously the silicon is distributed in the analyzed cross section.

In the +7 wt.% (Figure 9B) and +15 wt.% AlSi10Mg (Figure 9C) modification, some melt pool boundaries are more pronounced than others. This shows that the silicon is not equally distributed. Furthermore, in the +15 wt.% AlSi10Mg modification (Figure 9C), a size 72 µm indication is highlighted where the silicon signal is increased. It is hypothesized that the indication is a not fully dissolved AlSi10Mg powder particle.

In the base alloy, an increased silicon signal is measured at the location of the solidification cracks. During the polishing process, the SiO_2_-rich polishing emulsion accumulates within the solidification cracks. This leads to an increase in the signal. Therefore, the results close to cracks should be disregarded. Similar behavior is observed for lack of fusion and pores.

The qualitative EDX measurements suggest that no pronounced segregation is present in the modifications.

## 4. Discussion

### 4.1. Mechanisms to Reduce Solidification Cracks

In fusion welding, solidification cracks can be reduced by *(i)* controlling the solidification structure and *(ii)* using favorable welding conditions [18]. In this study, these mechanisms were investigated (*i)* by adding supplement powder and *(ii)* changing the process parameters.

In Figure 3, the base alloy was processed with varying process parameters with no base plate heating. It is expected that, with an increase in *VED*, the temperature gradient and cooling rate decreased [18], which leads to reduction in the solidification crack susceptibility, according to Rappaz et al. [38]. Only a minor change in the solidification crack frequency was observed in Figure 3. Overall, varying *VED* = 52 J/mm^3^ and 125 J/mm^3^ could not lead to a significant reduction in the solidification crack length and frequency.

In comparison, Zhou et al. [13] found that, at *VED* = 224 J/mm^3^ and 513 J/mm^3^ (*P* = 200 W and 350 W, *v* = 100 mm/s and 400 mm/s, *t* = 30 µm, *h* = 130 µm), no cracks were detected for the EN AW-5083 (AlMg4.5Mn0.7) alloy. However, Zhou et al. [13] measured a significant reduction in the magnesium concentration, due to a disproportionate evaporation during the LPBF process, as well as densities between 93% and 99% at *VED* = 224 J/mm^3^ and 513 J/mm^3^. Furthermore, at higher scan velocities, *v* > 400 mm/s solidification cracks could not be avoided. In conclusion, Zhou et al. [13] showed that processing EN AW-5083 without solidification cracks is possible, at the cost of (a) changing the magnesium concentration from 3.27 wt.% in the powder to about 1.5 wt.% in the specimen, (b) lower densities, and (c) slower build up rates (*v* < 400 mm/s). Even though solidification cracks were avoided with *VED* = 224 J/mm^3^ and 513 J/mm^3^, the LPBF-produced specimens cannot be specified as EN AW-5083 anymore, due to the disproportionate evaporation of magnesium. It is concluded that *(i)* adjusting the parameters *P*, *v*, and *d*_f_ to reduce the solidification cracks is not a valid strategy to eliminate solidification cracks in EN AW-5083 or AA5083. This agrees with the findings of this study.

For the experiments shown in Figure 5 and Figure 6, the base alloy is supplemented with +7 wt.% and +15 wt.% AlSi10Mg. The modifications eliminate the solidification cracks in the complete process window. Kouraytem et al. [43] showed that pores, similar to the ones present in Figure 5 and Figure 6, can cause solidification cracks. Therefore, the results suggest that the benefits of supplementing AlSi10Mg cannot be decreased by the pores observed in these cross sections. The results suggests that the benefit of supplementing AlSi10Mg is independent of the combination of the process parameters. Furthermore, the findings suggest that eliminating solidifications cracks in LPBF is a metallurgical problem. This allows to further adjust the combination of process parameters to avoid the common processing defects, such as pores, lack of fusion, and even the change in the magnesium concentration, as shown by [13], in the supplemented base alloy without expecting solidification cracks.

As it is known from fusion welding, adding silicon leads to a low melting point phase that reduces the solidification crack susceptibility [18,44]. Furthermore, it was found that supplementing AlSi10Mg increases the grain refinement and texture. With supplementing +7 wt.% AlSi10Mg, the melt pool boundaries are visible and restrict the grains to overgrow multiple layers, as can be seen in Figure 7. In this experiment, the grain refinement effect was saturated already at +7 wt.% AlSi10Mg and mean grain diameter is reduced by 40% relative to the base alloy (Table 7). It is hypothesized that either all the nucleation particles are already activated or the process parameters are limiting further grain refinement. In summary, supplementing AlSi10Mg increases the grain growth restriction [20].

Zhou et al. [13] produced a pre-alloyed powder alloy with an addition of 0.7 wt.% zirconium to AA5083. This leads to a significant grain refinement of 90%, relative to the base alloy mean grain size (Table 7). Mehta et al. [32] showed that this approach is also applicable for AA6061. Martin et al. [34] added zirconium nano particles to the powder alloy to promote grain refinement and eliminate the solidification cracks in AA7075. These findings further support the hypothesis that solidification cracks are a metallurgical problem.

Modifying a base alloy with a supplement powder, such as AlSi10Mg, reduces the solidification cracks because of two mechanisms. First, the grain refinement is increased and, second, a low melting-point silicon rich phase is added.

### 4.2. Feasibility of the Mixing Strategy

A homogeneous microstructure leads to homogeneous material properties. Therefore, the added silicon from the AlSi10Mg is preferably distributed homogeneously, so that all solidification cracks are eliminated. Figure 9 depicts the qualitative EDX measurements. The qualitative EDX measurements suggest that there is a segregation present. However, overall, the silicon is distributed sufficiently enough because the solidification cracks were avoided in all cross sections, as shown in Figure 5 and Figure 6. Adding a silicon-rich supplement powder to an aluminum alloy was already done successful in previous studies: Aversa et al. supplemented 50 wt.% AA7075 with 50% AlSi10Mg [25], and Montero-Sistiaga et al. supplemented up to 4 wt.% elemental silicon to AA7075 [24].

In conclusion, it is a feasible process strategy to supplement the base alloy by manually mixing it with AlSi10Mg, in order to eliminate solidification cracks. As shown by Skelton et al. [30], the size distribution of the metal powders can have an effect on the homogeneity of the blend. However, some questions still remain unanswered. What is the influence of the mixing strategy on the material properties? What are the limits of mixing that lead to an inhomogeneous silicon distribution? These questions are known in the field of particuology and addressed in [45]. The influence of the mixture procedure on the quality of the part, namely the porosity, lack of fusion, or the segregation, should be addressed and researched in-depth.

## 5. Conclusions

Solidification cracks hinder the development of new aluminum alloy powders for LPBF. In this study, a practical approach to overcome solidification cracks was presented. Up to now, there is no known experimental data on supplementing AlSi10Mg powder to the base alloy powder EN AW-5083 (AlMg4.5Mn0.7). Most research focusses on the aluminum alloy powder AA7075.

It was possible to eliminate the solidification cracks by supplementing +7 wt.% AlSi10Mg powder to EN AW-5083 powder. The powders were mixed before the specimens were manufactured with LPBF. The microstructural analysis showed that supplementing AlSi10Mg leads to a grain refinement of 40%, relative to the mean grain size of the base alloy. Subsequently, the grain refinement caused by supplementing AlSi10Mg helps to eliminate the solidification cracks, besides the known effect of adding a silicon-rich phase.

By varying the process parameters between *VED* = 52 J/mm^3^ and 125 J/mm^3^, the solidification cracks in the base alloy EN AW-5083 could not be eliminated. The results suggest that eliminating solidification cracks in EN AW-5083 during LPBF is a metallurgical problem and requires a modification of the base alloy.

In literature, pre-alloying or adding nanoparticles are both procedures that are used to avoid solidification cracks. In comparison to those, the presented approach is considered practical because of the availability of AlSi10Mg as a supplementing powder. The approach can be easily transferred to other powder alloys and supplement powders.

Furthermore, this study highlights the mechanisms to eliminate the solidification cracks, which are prerequisites for modelling the phenomenon. Additionally, this study provides valuable experimental data, which can also be used for modelling and calculating the required amount of supplement powder. Numerous models are available to describe the solidification crack susceptibility. They can be used as a starting point for computer-aided alloy design.

## Figures and Tables

**Table 1:** Alloy composition of EN AW-5083 (AlMg4.5Mn0.7), according to DIN EN 573-3 [41].

	Si (wt.%)	Fe (wt.%)	Cu (wt.%)	Mn (wt.%)	Mg (wt.%)	Cr (wt.%)	Al (wt.%)
EN AW-5083 (AlMg4.5Mn0.7)	0.4	0.4	0.1	0.4–1.0	4.0–4.9	0.05–0.25	Balance

**Table 2:** Applied process parameters for the LPBF experiments. For all experiments, no base plate heating was used, and the layer thickness was kept constant at 30 µm.

	Laser Power *P* (W)	Scan Velocity *v* (mm/s)	Focal Diameter *d_f_* (µm)	Hatch Distance *h* (µm)	Volumetric Energy Density *VED* (J/mm^3^)
A	150	500	100	80	125
B	250	1000	100	80	104
C	250	1500	100	80	69
D	250	500	200	150	111
E	325	1000	200	150	72
F	350	1500	200	150	52

**Table 3:** Alloy composition of the base alloy, supplement AlSi10Mg powder, and two modifications.

	Si (wt.%)	Mg (wt.%)	Mn (wt.%)	Al (wt.%)	Source
EN AW-5083 (AlMg4.5Mn0.7)	0.14	4.0	0.67	Balance	Data sheet
AlSi10Mg	9.84	0.32		Balance	Data sheet
MOD1: +7 wt.% AlSi10Mg	0.82	3.74	0.62	Balance	Calculated
MOD2: +15 wt.% AlSi10Mg	1.60	3.45	0.57	Balance	Calculated

**Table 4:** Results of the density measurements of the EN AW-5083 specimens, done with image processing. The process parameters used are listed in Table 2.

Process Parameter	Density (%)
A	97.49
B	96.03
C	96.92
D	97.39
E	96.88
F	95.44

**Table 5:** Results of the density measurements of Modification I specimens, done with image processing. The process parameters used are listed in Table 2.

Process Parameter	Density (%)
A	97.67
B	95.26
C	99.11
D	98.56
E	98.31
F	98.96

**Table 6:** Results of the density measurements of Modification II specimens, done with image processing. The process parameters used are listed in Table 2.

Process Parameter	Density (%)
A	98.51
B	96.60
C	98.61
D	99.10
E	98.89
F	97.61

**Table 7:** Calculated mean grain diameter from the EBSD results for the different alloys.

Alloy	Mean Grain Diameter (µm)	Source
AlMg4.5Mn0.7	69	This study.
+7 wt.% AlSi10Mg	41	This study.
+15 wt.% AlSi10Mg	39	This study.
AlMg4.5Mn0.7	82	[13]
+0.7 wt.% Zr	7	[13]

## Data Availability

The data presented in this study are available on request from the corresponding author.
